# Engaging Pediatric Intensive Care Unit (PICU) clinical staff to lead practice improvement: the PICU Participatory Action Research Project (PICU-PAR)

**DOI:** 10.1186/1748-5908-9-6

**Published:** 2014-01-08

**Authors:** Jean-Paul Collet, Peter W Skippen, Mir Kaber Mosavianpour, Alexander Pitfield, Bubli Chakraborty, Garth Hunte, Ronald Lindstrom, Niranjan Kissoon, William H McKellin

**Affiliations:** 1 Department of Pediatrics, University of British Columbia, 4480 Oak Street, Vancouver, British Columbia, Canada; 2 British Columbia Children’s Hospital, 4480 Oak Street, Vancouver, British Columbia, Canada; 3 Child and Family Research Institute, 950 West 28th Avenue, Vancouver, British Columbia, Canada; 4 Pediatric Intensive Care Unit, BC Children’s Hospital, 4480 Oak Street, Vancouver, British Columbia, Canada; 5 Department of Anthropology, University of British Columbia, 6303 NW Marine Drive, Vancouver, BC, Canada; 6 Emergency Department, Saint Paul’s Hospital and Department of Emergency Medicine, University of British Columbia, 1081 Burrard Street, Vancouver, British Columbia, Canada; 7 Provincial Health Services Authority, 1380 Burrard Street, Vancouver, British Columbia, Canada; 8 School of Leadership Studies, Royal Roads University, 2005 Sooke Road, Victoria, British Columbia, Canada

**Keywords:** PICU, ICU, Quality improvement, Community of practice, Participatory action research, Distributed leadership, Engagement, Learning community, Reflective practice, Children

## Abstract

**Background:**

Despite considerable efforts, engaging staff to lead quality improvement activities in practice settings is a persistent challenge. At British Columbia Children’s Hospital (BCCH), the pediatric intensive care unit (PICU) undertook a new phase of quality improvement actions based on the Community of Practice (CoP) model with Participatory Action Research (PAR). This approach aims to mobilize the PICU ‘community’ as a whole with a focus on practice; namely, to create a ‘community of practice’ to support reflection, learning, and innovation in everyday work.

**Methodology:**

An iterative two-stage PAR process using mixed methods has been developed among the PICU CoP to describe the environment (stage 1) and implement specific interventions (stage 2). Stage 1 is ethnographic description of the unit’s care practice. Surveys, interviews, focus groups, and direct observations describe the clinical staff’s experiences and perspectives around bedside care and quality endeavors in the PICU. Contrasts and comparisons across participants, time and activities help understanding the PICU culture and experience. Stage 2 is a succession of PAR spirals, using results from phase 1 to set up specific interventions aimed at building the staff’s capability to conduct QI projects while acquiring appropriate technical skills and leadership capacity (primary outcome). Team communication, information, and interaction will be enhanced through a knowledge exchange (KE) and a wireless network of iPADs.

**Relevance:**

Lack of leadership at the staff level in order to improve daily practice is a recognized challenge that faces many hospitals. We believe that the PAR approach within a highly motivated CoP is a sound method to create the social dynamic and cultural context within which clinical teams can grow, reflect, innovate and feel proud to better serve patients.

## Background

National reports have highlighted undesirable practice variations leading to sub-optimal healthcare, medical errors and adverse patient outcomes [1-6]. Numerous interventions to standardize practice have been developed based on well-established theories and techniques [7-11]. However, despite considerable efforts nationwide, the change in practice quality has been ‘frustratingly low’ [6], and staff engagement in practice settings to lead quality improvement activities is a persistent challenge.

Over the past decade, the Pediatric Intensive Care Unit (PICU) at British Columbia Children’s Hospital (BCCH), as member of the Canadian Pediatric Critical Care Collaborative, experienced a track record of successes following the quality improvement approach with repeated Plan-Do-Study-Act (PDSA) cycles. In 2009, the Provincial Health Services Authority, which governs BCCH, adopted the LEAN methodology [12-18] in an effort to improve quality and efficiency to the overall healthcare system. LEAN Leaders and the PICU team completed 23 Rapid Process Improvement Workshops (RPIWs) over four years. While the initial efforts were impressive, longer-term success was mixed because gains from only 9 of 23 RPIW projects (about 40%) have been sustained at one year. The reason was attributed in part to the way RPIW projects were implemented with unit and organizational leadership selecting isolated component deficiencies without seeking input and engagement of the broad team of care providers and staff.

Considering the complexity of the PICU environment, a third phase of QI was conceived based on the Community of Practice (CoP) model with Participatory Action Research (PAR) [19-26] to support reflection, learning and innovation in everyday work [23-26].

### Rationale

The reason for this approach is based on the premise that in complex adaptive systems [27-35] such as the PICU, one approach to promote changes consists of mobilizing the community as a whole with a focus on practice; namely, to create a CoP [23,24]. Within our CoP, we aim to create the conditions of a collaborative, reflective and innovative experiential system [36] that will enable collective discussions around daily practice issues and finding solutions for improvement by integrating tacit-explicit knowledge [20]. Further, there is a growing body of literature that supports the active engagement of all staff when undertaking change management initiatives in hospitals, particularly in relation to patient safety and quality [35,37,38]. As Lindstrom suggests, ‘front-line ownership of the problems and, more importantly, collective solutions has highlighted the importance of and effectiveness of distributed leadership’ [37]. And when it concerns staff engagement, Van de Ven observes that there are three key principles regarding the gap between what the theory says and what actually happens in practice [39]:

1. Translating knowledge into practice requires a much better understanding of how to engage stakeholders and communicate across stakeholder knowledge boundaries;

2. Scientific knowledge and practical knowledge are distinct, and that a more pluralist view is required, one that allows complementary perspectives to understand reality;

3. Knowledge production is a problem because it has reflected scientific inquiry that has often not engaged stakeholders other than researchers; thus, the research is not appropriately grounded in day- to-day realities and generates little impact.

Consequently, there is a pressing need for staff engagement. Consistent with a PAR approach, this involves engaging and working with, not on or for, clinical staff – those closest to and on the frontline of healthcare.

### PAR approach

PAR is comprised of three basic elements [20,40]:

1. Participation broadens who participates in the research process [22], in this case multiple stakeholders comprised of researchers, clinical and managerial decision-makers, and also patients/family members;

2. Action, which is emphasized over just generating new knowledge [22];

3. Research focuses on perspectives locally defined by, *e.g*., decision-makers; shares power between researchers and decision-makers; expands the purview of knowledge generation from academia to the community; and realigns the researchers’ role from directing to facilitating the process [22].

This set of elements constitutes the collaborative, collective nature of PAR. As Greenwood and Levin assert, this is ‘co-generative inquiry because it is built on professional researcher-stakeholder collaboration and aims to solve real-life problems in context. Co-generative inquiry processes involve trained professional researchers and knowledgeable local stakeholders who work together to define the problems to be addressed, to gather and organize relevant knowledge and data, to analyze the resulting information, and to design social change interventions. Together these partners create a powerful research team’ ([41] p. 54). Unlike traditional research, PAR deliberately intervenes in the research setting [21]. This is an important distinction. However, scholars such as Greenwood and Levin caution against PAR as a short-term intervention; rather, it is a ‘continuous and participative learning process’ ([20] p.18). This is an important point, which is explicitly factored into our study and the continuous nature of QI and which underscores the important role of CoPs.

### Research questions

Our PAR approach addresses four research questions:

1. How do frontline clinical staff and decision-makers identify and conceptualize improving the quality of care a patient receives?

2. Currently, in what ways do frontline clinical staff engage in QI activities?

3. What resources, supports, enablers and capacity are required for frontline clinical staff to increase their engagement in QI to lead and conduct projects?

4. What specific strategies are required to address the needs identified by frontline clinical staff?

### Overall research goal

To sustain success in improving everyday practice in the PICU by building and supporting staff’s individual and collective capacity to conduct QI projects in an environment of distributed leadership.

### Specific objectives

1. To determine frontline clinical staff’s engagement, collective capacity and ability to conduct projects for daily practice improvement.

2. To develop and support staff’s collective engagement, ability and leadership to improve practice.

3. To ensure sustainability of the change within the context of a new PICU culture of distributed leadership.

4. To generate a PAR practice QI framework over the course of the study.

### Design

**Figure 1:**
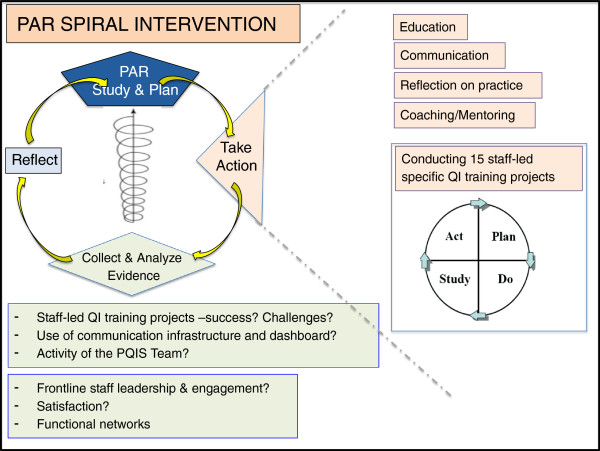
PICU-PAR project description – PAR spiral and specific interventions.

**Figure 2:**
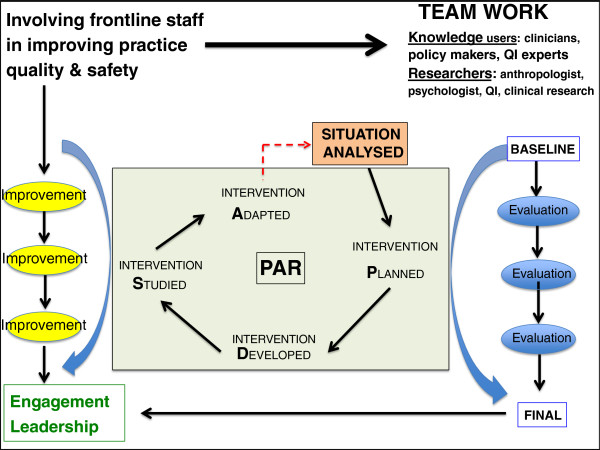
PAR description with teamwork process and reflection for improved adapted intervention.

**Figure 3:**
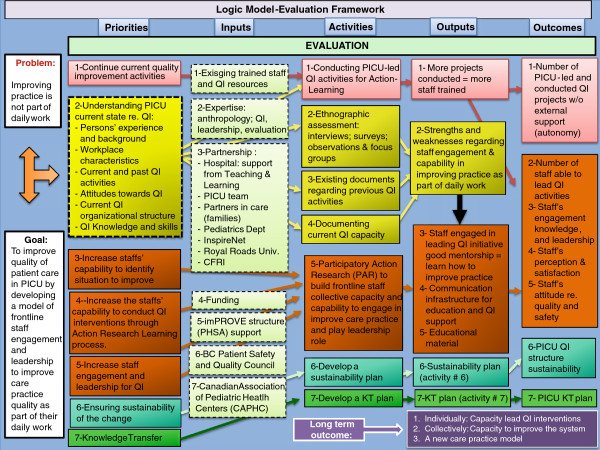
Logic Model for PICU-PAR project evaluation framework.

An iterative two-stage PAR process employing mixed methods will be used to explore the PICU environment and to implement specific interventions within the context of an emerging CoP. The overall research approach is schematically illustrated in Figures 1 and 2 with the evaluation framework presented in Figure 3.

### Stage 1 Describe the collective action of the PICU using artifacts, perceptions and patterns in order to offer an account of action in everyday practice

We will be employing several strategies during the observational phase of the study that will provide us with an ethnographic description of the practice of care. We will be enquiring into the experiences and perspectives of clinical staff around bedside care and quality endeavors in the PICU. Contrasts and comparisons across participants, time and activities, will help understanding PICU culture and experience. The mixed methods for PICU system description include both qualitative and quantitative methods:

### Qualitative methods

#### *Observation*

We will observe communication and patterns of interaction among the PICU staff, patients, and their parents [42-44] and evaluate effects of these patterns on the staff’s ability to engage and lead during routine activities such as handover rounds and the orientation of new staff. All QI activities conducted in the past three years will be reviewed and described with regard to the type of project, initiator, external support, staff involvement, output and sustainability. This information will be discussed with staff as a way to engage discussion about improvement and changes.

### Interviews

Individuals and groups will be engaged in semi-structured interviews that will enable staff to contextualize the issues of quality improvement with concrete examples from their own patterns of practice. Some families will also be interviewed in regard to communication with staff and to obtain their perception of care quality. The interviews will also address the issue of ‘readiness to change’ [45-47] to inform optimal choice of possible interventions [48]. Finally, during the semi-structured interviews, participants will be asked to diagram their own networks of individuals whom they recognize as leaders in different areas of quality improvement. This will contribute to the quantitative analysis of social networks within the PICU [49-53] and help in developing future interventions strategies.

### Focus groups

The dynamic aspect of group discussions will be used to identify solutions. One set of focus groups will be multidisciplinary, composed of individuals representing different disciplines, while the second set will be discipline specific. At least one focus group will be conducted with families.

### Quantitative methods

#### *Staff engagement survey*

Engagement will be assessed using the ‘Employee Engagement Questionnaire’ [54], a validated tool that has been widely used to assess staff engagement [54], including on a yearly basis in the PICU for the past three years.

### Staff leadership surveys

Frontline staff leadership will be assessed with two instruments.

1. The ‘Healthcare Team Vitality Instrument’ (HTVI): This is a short survey developed by the Institute of Healthcare Improvement as part of the initiative Transforming Care at Bedside. It measures team vitality with an emphasis on front-line staff empowerment and engagement, perception of a work environment, supportive, effective communication, and team collaboration. The HTVI has been validated [55] and is widely used in North America, as it has been in our PICU during the last three years, therefore providing a good reference point against which changes can be assessed.

2. Leadership abilities: Leadership capacity will be assessed by employing a questionnaire in conjunction with the LEADS in a Caring Environment framework [56], which is being used by BCCH to assess leadership abilities and improvement during specific interventions.

### Network analysis surveys

Social network analysis will be used to identify existing networks in the unit. Social network information is important to understand the unit’s functioning, patterns of communication, and for planning effective interventions to support organizational changes at a later stage [57,58]. Network analysis can also be repeated over the course of the project to assess changes over time [49-53,57].

### Stage 1 Sampling

All PICU staff will be invited to participate. Surveys will be offered to everyone, while personal interviews and focus group discussions will identify specific sub-groups based on categories of personnel and seniority in the unit. Ethnographic observations and network analysis will also require specific sampling strategies.

### Stage 1 data analysis

1. Observation: Audio transcripts (and possibly video recordings) will be analyzed using conversational analysis methods [50,59] to identify the distribution of knowledge and collaboration among members of the staff [60], to augment topics for qualitative interviews, and as a vehicle for reflection and self-assessment [44,61-63].

2. Interviews and focus groups: The semi-structured interviews will be transcribed, and entered into ATLAS-ti, a qualitative database for coding. We will employ techniques from conversational analysis [59,64] and cognitive linguistic discourse analysis [65] to describe the themes and identify information schemas in the interview data [66]. The analysis process will be continuous; new data will be compared to existing ones to identify emerging information (themes or ideas) with the ultimate objective to create a body of knowledge regarding the PICU attitudes and thoughts. The interview iterative process will continue until saturation is reached, defined as no new information or themes about a specific topic in three consecutive interviews [67-69], and clear assertions can be made regarding the topic studied. For focus groups, the analysis will also consider the dynamics of the discussions and the role of the individuals.

3. Survey questionnaires: Because one important objective is communicating results to study participants and decision-makers, special attention will be given to descriptive statistics, using point estimates and 95% confidence intervals, as well as frequency tables and graphs.

4. Social network analysis: We will use UCINET 6 [70] to illustrate the different relational and structural measures. Sociograms, other forms of network diagrams, and matrices will depict the relationship and structure of social and informational networks on the unit. Before-after intervention will be contrasted with regard to the type of measures identified.

### Stage 2 The PAR spirals

#### *Building the communication structure*

One specific consideration is the development of a positive environment to facilitate and encourage reflections about actual care practices and possible improvement. With appropriate coaching and support, a CoP can be expected to develop progressively [24,71], enriched by the participation of all PICU members who will bring their specific insight on practice and improvement. Two staff members who have extensive expertise in this area will lead a ‘PICU QI Support Team’, which will be a structuring element to guide and support the CoP’s functions. Intervention in the context of PAR will also benefit from a solid knowledge exchange (KE) that facilitates communication among frontline staff, decision-makers, quality leaders and researchers in a continuous, multi-directional way. A web-based dashboard will visually describe the PAR project’s status and the evolution of each QI change project. Periodic reports, posters and newsletters will circulate and remain posted on the study dashboard to facilitate discussions with the whole team. The acquisition of iPAD tablets will enable flexible access to the study dashboard and create new communication streams with nurses who primarily stay at the patient’s bedside. The iPAD tablets will also be used for data collection during quality change interventions – with automated generation of run charts and control charts when new data are entered.

### Identifying and implementing specific QI interventions

Specific interventions will be incorporated according to the needs/gaps identified during Stage 1 and contextual factors. The main educational/training features will be ‘action learning’ [72] through engaging PICU staff leading their own QI change interventions. With appropriate coaching, mentoring and communication, we expect care providers both to individually engage in everyday practice improvement, and collectively lead changes toward a new emerging practice culture. Webinars and educational documents will be stored in a library with remote access through the study dashboard.

### Continuous evaluation and reflection

Continuous evaluation is an integral part of a PAR to refine, modify and adjust the specific interventions as per action research principles [25,26]. Staff capabilities, skills, leadership, and engagement will be assessed regularly, with a main review conducted every six months to guide the PAR cycles. Relevant performance metrics will be used to visualize results and share the information with staff. New interventions will then be identified and refined for a new PAR spiral.

### Project outcomes

The different outcomes are structured according to the project’s evaluation framework presented in Figure 3. The following is a list of key areas that are expected to show effects, with related indicators.

1. PICU frontline staff’s current state and actual functioning with regard to quality improvement: QI definition and model used; QI practice and experience; Existing QI knowledge and support; Perceived barriers and facilitators for QI.

2. Capacities developed to support frontline staff to engage and lead actions to improve daily practice: Development of educational and training materials as well as an interactive communication structure through the study dashboard; Development of new PICU and hospital policies that encourage and value staff’s reflections about care practice and improvement.

3. New frontline staff capabilities to initiate and conduct practice improvement interventions: Staff engagement in QI initiatives will be assessed through the following: number of projects developed and completed by bedside clinicians, changes in engagement and leadership survey scores, and number of visits to the quality dashboard. The ultimate goal of these changes is to affect the quality of patient care, but this outcome cannot be measured within a three-year span.

4. Frontline staff capabilities and willingness to become leaders in reflective practice: PICU staff self-efficacy and self-confidence leading changes as well as satisfaction with the new culture of distributed leadership will be assessed through interviews and focus groups.

### Analysis of the impact

The CoP-PAR impact will be assessed through qualitative and quantitative approaches assessing before-after changes in important outcomes (engagement, collective activities, leadership, and barriers). Factors associated with behavioral changes will be identified through multivariate modeling. The analysis will also consider the high inter-variables correlations and the numerous tests to prevent misinterpretation of the study results.

### Knowledge exchange (KE) and sustainability

During the course of the study, the KE and dashboard material will ensure rapid exchange of relevant information among PICU staff. On-going knowledge transfer activities will be planned for specific stakeholders, guided by Lavis’s questions [73], to inform them of the study results.

The main strength of our sustainability plan lies in a ‘Training the trainers’ process, with new QI experts supporting and training other staff. It also includes: keeping the KE website and dashboard active for communication support after the study; maintaining access to local support resources that remain through the project’s partners; organizing future coaching and leadership sessions to assist staff’s capacity to retain their skills and develop new ones.

### Ethics

University of British Columbia ethics review board has reviewed and given approval for the project. The main ethical issue was related to the need to clearly separate managers from clinical staff in organizing sampling and interviews. Although managers will help, they will not be involved in identifying and contacting frontline workers.

### Timeline and study status

**Figure 4:**
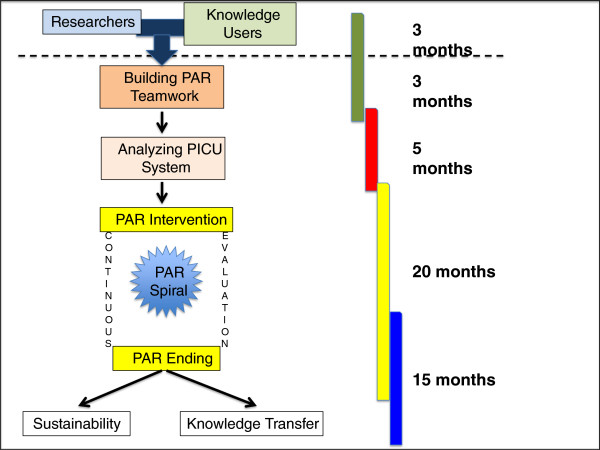
PICU-PAR Research Plan with stages and timeline.

This is a three-year project with six months for the initial PICU system description (phase 1) and the remaining for developing the PAR approach employing three to four spirals. During the last six months of the project, KE and sustainability will become a high priority. The project started in August 2012 with meetings of care providers, PICU managers, hospital executive and managers and researchers. Our methodology was developed over four months, and ethical approval was granted two months later. The initial interviews and surveys commenced April 2013. Figure 4 shows the study timeline.

## Discussion

Lack of staff leadership to improve daily practice is a recognized challenge that faces many hospitals. We believe that the PAR approach within active and motivated CoPs is a sound methodology to create the social culture and context within which clinical teams can grow, reflect, innovate and feel proud to better serve patients.

CoP is a recognized model for communities to learn and improve [23,24,74,75]. In particular, the sharing of experiences and ideas among CoP members is a source of reflection that enriches learning and subsequent changing practice [23,24,76].

However, two systematic reviews on CoP did not find strong evidence supporting its positive effect [77,78]. One reason may be the way in which CoPs generally develop and function with voluntary participation and informal communication around practical experience (social networks type); the primary objective being to improve healthcare, not to publish. Consequently, we do not yet know the actual effect of CoPs toward improving practice.

Our PAR approach enables the co-generation of practical data to facilitate future development. This is supported by different socio-cultural theories such as Situated Learning [24,79], Activity Theory [80-85], and Distributed Cognition [62,81], all linked to the broad concept of ‘Learning Communities’ and rooted to Vygotsky’s landmark work on social learning [84,85]. We expect that, in the context of PAR, the CoP will identify gaps, defects and challenges and will also generate innovative solutions to be implemented through a sequence of improvement spirals [25,26,86-88].

With this approach, we aim to better understand how to increase PICU frontline staff engagement and leadership to improve care, and to assess the overall impact of introducing a PAR process within a well-structured CoP. Throughout the study, we expect to obtain a deeper insight into the CoP development process, including KE and sustainability. Our goal is to create a PAR approach and framework that is generic enough to be used in other units yet flexible enough to meet the units’ characteristics and specific needs. If successful, we expect this framework to be adopted and refined in other units at BCCH and other pediatric centres across Canada.

Developing the PICU PAR approach was also a learning experience for all participants. It is the product of a multidisciplinary team of clinicians, managers, policy-makers, and researchers who all desire to actively participate. Stakeholder commitment constitutes the foundation on which PAR can emerge; it requires participants to dedicate time, to be open-minded to understand/accept the different points of view, to accept a degree of uncertainty and to be interested in the process. Stakeholder commitment, therefore, can be taken as a sign of interest and trust in the project and mutual respect among team members. From our experience, we see the PAR development process as a unique opportunity to strengthen the PICU team.

## Abbreviations

BCCH: British Columbia children’s hospital; CoP: Community of practice; HTVI: Healthcare team vitality instrument; KE: Knowledge exchange; PAR: Participatory action research; PICU: Pediatric intensive care unit; PDSA: Plan-do-study-act; PHSA: Provincial health services authority; RPIW: Rapid process improvement workshops.

## Competing interests

No competing interests to declare.

## Authors’ contributions

JPC WM PS MKM AP initially developed the concepts with contributions from BC GH RL NK. JPC wrote the initial draft with contributions from all authors during the editing. All authors provided comments and approved the final version.
